# Enabling Adherence to Treatment (EAT): a pilot study of a combination intervention to improve HIV treatment outcomes among street-connected individuals in western Kenya

**DOI:** 10.1186/s12913-023-10215-1

**Published:** 2023-11-30

**Authors:** Mia Kibel, Monicah Nyambura, Lonnie Embleton, Reuben Kiptui, Omar Galárraga, Edith Apondi, David Ayuku, Paula Braitstein

**Affiliations:** 1 MD Program, Temerty Faculty of Medicine, University of Toronto, Toronto, ON Canada; 2 Academic Model Providing Access to Healthcare (AMPATH), P.O. Box 4606-30100, Eldoret, Kenya; 3 Department of Global Health and Health System Design, Icahn School of Medicine Mount Sinai, New York, NY USA; 4 Department of Health Services Policy and Practice, and International Health Institute, Brown University School of Public Health, Providence, RI USA; 5 Department of Child Health and Paediatrics, College of Health Sciences, Moi University, Eldoret, Kenya; 6 Department of Mental Health and Behavioral Sciences, School of Medicine, College of Health Sciences, Moi University, Eldoret, Kenya; 7 Department of Epidemiology and Medical Statistics, School of Public Health, College of Health Sciences, Moi University, Eldoret, Kenya

**Keywords:** Homeless persons, Homeless youth, HIV, Kenya, Antiretroviral therapy

## Abstract

**Background:**

Street-connected individuals (SCI) in Kenya experience barriers to accessing HIV care. This pilot study provides proof-of-concept for Enabling Adherence to Treatment (EAT), a combination intervention providing modified directly observed therapy (mDOT), daily meals, and peer navigation services to SCI living with HIV or requiring therapy for other conditions (e.g. tuberculosis). The goal of the EAT intervention was to improve engagement in HIV care and viral suppression among SCI living with HIV in an urban setting in Kenya.

**Methods:**

This pilot study used a single group, pre/post-test design, and enrolled a convenience sample of self-identified SCI of any age. Participants were able to access free hot meals, peer navigation services, and mDOT 6 days per week. We carried out descriptive statistics to characterize participants’ engagement in EAT and HIV treatment outcomes. We used McNemar’s chi-square test to calculate unadjusted differences in HIV outcomes pre- and post-intervention among participants enrolled in HIV care prior to EAT. We compared unadjusted time to initiation of antiretroviral therapy (ART) and first episode of viral load (VL) suppression among participants enrolled in HIV care prior to EAT vs. concurrently with EAT using the Wilcoxon rank sum test. Statistical significance was defined as *p* < 0.05. We calculated total, fixed, and variable costs of the intervention.

**Results:**

Between July 2018 and February 2020, EAT enrolled 87 participants: 46 (53%) female and 75 (86%) living with HIV. At baseline, 60 out of 75 participants living with HIV (80%) had previously enrolled in HIV care. Out of 60, 56 (93%) had initiated ART, 44 (73%) were active in care, and 25 (42%) were virally suppressed (VL < 1000 copies/mL) at their last VL measure in the 19 months before EAT. After 19 months of follow-up, all 75 participants living with HIV had enrolled in HIV care and initiated ART, 65 (87%) were active in care, and 44 (59%) were virally suppressed at their last VL measure. Among the participants who were enrolled in HIV care before EAT, there was a significant increase in the proportion who were active in HIV care and virally suppressed at their last VL measure during EAT enrollment compared to before EAT enrollment. Participants who enrolled in HIV care concurrently with EAT had a significantly shorter time to initiation of ART and first episode of viral suppression compared to participants who enrolled in HIV care prior to EAT. The total cost of the intervention over 19 months was USD $57,448.64. Fixed costs were USD $3623.04 and variable costs were USD $63.75/month/participant.

**Conclusions:**

This pilot study provided proof of concept that EAT, a combination intervention providing mDOT, food, and peer navigation services, was feasible to implement and may support engagement in HIV care and achievement of viral suppression among SCI living with HIV in an urban setting in Kenya. Future work should focus on controlled trials of EAT, assessments of feasibility in other contexts, and cost-effectiveness studies.

**Supplementary Information:**

The online version contains supplementary material available at 10.1186/s12913-023-10215-1.

## Background

The United Nations Human Rights Council identifies homelessness as a “global human rights crisis,” driven by inequalities in wealth and land access [1]. Although definitions vary, international bodies recognize that homelessness extends beyond not having a house [1–4]. Homelessness may be included within the broader experience of being street-connected, where as a result of insecure or inadequate housing, the street plays a central role in an individual’s everyday life or identity [1, 5, 6]. Street-connected individuals (SCI) include adults, children, and adolescents who may be unsheltered or inadequately sheltered (sleeping in a temporary or unsafe location), and may be part of a street-based community or economy, for example, for drug use or sex work.

Across varied global contexts, SCI experience profound violations of their right to health and right to life, with high burdens of morbidity and mortality from infectious and chronic disease, mental illness, substance use, and violence [4, 7–10]. For example, research in high-, middle-, and low-income settings shows high rates of HIV among street-connected adults [4, 8, 11] and youth [7, 12–14]., In low- and middle-income settings, including sub-Saharan African nations with highly generalized HIV epidemics, drivers of HIV risk among SCI include age, gender, employment, substance use, sexual and gender-based violence, and survival sex (sex in exchange for food, shelter, or other material necessities) [7, 11, 12]. The scope of the challenge to address HIV risks among SCI is enormous: up to 238 million people in sub-Saharan Africa are homeless or inadequately housed, including a large number of children and youth [15].

SCI in sub-Saharan Africa experience social and structural barriers to accessing HIV treatment, including discrimination, stigmatization, criminalization, and poverty [1, 16–19]. SCI are also likely to experience food insecurity and to lack a safe place to take medications [20–22]. Thus, it is not surprising that the limited available research suggests that SCI in sub-Saharan Africa have poor HIV treatment outcomes [23]. For example, a 2019 intervention in urban western Kenya that connected SCI under 30 years of age to HIV testing and treatment found that less than one-third of participants achieved viral suppression over two years of follow up [23]. This suggests that even in the context of an intervention to support treatment, the rate of viral suppression among SCI is less than half the rate of viral suppression among the local general population [24]. Given the multiple inequities that limit SCI’s access to healthcare and HIV treatment, SCI are likely to benefit from interventions that combine behavioral and biomedical supports and address structural barriers to HIV treatment [25]. However, the majority of research on improving HIV treatment outcomes among SCI is based in high-income settings [26, 27]. There is an urgent need for evidence to guide the design and implementation of interventions that improve HIV treatment outcomes among SCI in sub-Saharan Africa.

One potential strategy for supporting HIV treatment among SCI in sub-Saharan Africa involves modified Directly Observed Therapy (mDOT). mDOT is an intervention originally developed to promote adherence to tuberculosis treatment, where all or some doses of medication are taken under direct supervision by a healthcare professional [28]. mDOT interventions have been adapted to support adherence to antiretroviral therapy (ART) for HIV, and have been shown to be particularly effective in populations with high rates of non-adherence [28, 29]. In addition to supervised dosing, mDOT may address other barriers to ART adherence by providing SCI with a safe care environment, a secure place to store medications, and facilitating regular contact with healthcare providers. mDOT has also been combined with other evidence-based strategies to support adherence to treatment in resource-constrained settings, including peer support and food [29]. An mDOT intervention has not yet been piloted with SCI in an urban setting in Kenya.

This pilot study describes the design, implementation, costs, and HIV outcomes of the Enabling Adherence to Treatment (EAT) intervention, a combination intervention providing mDOT and a daily meal alongside pre-existing peer navigation services, to SCI in an urban setting in western Kenya. The goal of this pilot study is to provide proof of concept for the use of the EAT intervention to support engagement in HIV care and improve rates of viral suppression among SCI in urban settings in western Kenya.

## Methods

### Study aim and design

This pilot study used a single group, pre/post-test design to provide proof of concept for EAT, a novel combination intervention among SCI in an urban setting in Kenya [30]. Proof of concept (also called proof of implementation) research is a form of Implementation Science research often conducted via pilot studies, which focuses on (1) generating evidence that a proposed intervention is feasible, i.e. can actually work in a real-life setting and (2) identifying factors to guide implementation of controlled trials [30–32]. EAT combined the following interventions:

- Biomedical: accessible ART and pre- and post-exposure prophylaxis for HIV
- Behavioral: modified directly observed treatment with doses observed by a pharmacy technologist, and peer navigation services to encourage engagement in care including follow-up for missed visits
- Structural: daily meals for participants

### Setting

The pilot study of the EAT intervention took place at the Rafiki Centre for Excellence in Adolescent Health at the Moi Teaching and Referral Hospital (MTRH) in Eldoret, Kenya. Eldoret has a population of 475,716 and is located in Uasin Gishu county [33]. Eldoret is home to the Moi University College of Health Sciences, MTRH, and is the headquarters of the Academic Model Providing Access to Healthcare (AMPATH) program. In partnership, AMPATH, the Kenyan Ministry of Health, Moi University, and MTRH provide HIV care for over 150,000 people across nearly 500 facilities and provide HIV testing, care and ART free of charge across western Kenya [34].

A 2016 count found that the population of SCI in Eldoret was approximately 1,900 individuals [12]. Although HIV prevalence in the general population of SCI is unknown, the 2016 HIV prevalence among SCI under 30 years of age was 2.7% among males and 8.9% among females, roughly consistent with the national HIV prevalence of 4% among males and 6.7% among females [12].

### Study population

SCI are defined as people who spend the majority of their time living and/or working on the streets, or for whom the streets play a central role in their everyday lives and social identities [5, 6]. SCI may include adults, youth, and children. They may be unsheltered or inadequately sheltered, and may be part of a street-based community or economy, for example, for drug use or sex work.

Originally, we planned to pilot the EAT intervention among street-connected children and youth only (because this pilot study was a sub-study of a larger project to engage street-connected youth in HIV care, see below). However, due to the ethical and practical challenges involved in providing food to some street-connected people living with HIV and not others, we expanded the study population to include individuals of any age who identified as street-connected.

### The Engaging Street Youth in HIV Interventions (ESYHI) study

This pilot study of the EAT intervention is a sub-study of the Engaging Street Youth in HIV Interventions Study (ESYHI). The goal of ESYHI is to identify, adapt, and pilot interventions to engage street-connected youth in Eldoret in the HIV prevention-care continuum. ESYHI study procedures are described in detail elsewhere [35]. In brief, the research team conducted a scoping literature review to identify HIV prevention and treatment interventions implemented successfully among populations at high risk of HIV in resource-constrained settings. The research team then conducted consensus-building activities, including “*mabaraza”* (traditional group discussions) with knowledge users including current and former street-connected youth, and volunteers and community members involved with street-connected youth. The research team and knowledge users identified mDOT with free daily meals as a potentially successful intervention to support HIV treatment among street-connected youth.

### Eligibility criteria

Individuals were eligible for inclusion if they: (1) self-identified as spending at least three-quarters of their time (including days and nights) on the street (defined as an area that is absolutely unsheltered or in an inadequate shelter like a “barracks,” an outdoor area with rudimentary covering*)* for at least the past three months, and (2) were receiving treatment for HIV or another condition requiring medication adherence, including tuberculosis, bacterial infections, pre-exposure prophylaxis (PrEP) or post-exposure prophylaxis (PEP) for HIV. Although we were primarily interested in ART for HIV, we chose not to limit eligibility to only individuals living with HIV in order to avoid de facto disclosure of HIV status, and to reduce the stigma associated with participating in EAT.

### Human subjects’ protections

This pilot study of the EAT intervention was reviewed and approved by the Moi University College of Health Sciences and MTRH Institutional Research Ethics Committee and the University of Toronto Research Ethics Board. Individuals aged 18 years or older who wished to participate provided written informed consent. Individuals under the age of 18 provided written informed assent themselves, and we sought guardian written informed consent. For participants under the age of 18 with no guardian, the Moi University College of Health Sciences and Moi Teaching and Referral Hospital Institutional Research Ethics Committee and the University of Toronto Research Ethics Board provided a waiver of guardian informed consent, and we obtained written informed consent from the senior social worker and written informed assent from the participants themselves. The Moi University College of Health Sciences and Moi Teaching and Referral Hospital Institutional Research Ethics Committee and the University of Toronto Research Ethics Board waived the need for informed consent for participants under the age of 18. Informed consent was obtained from all participants or their legal guardians, or a waiver of informed consent was obtained from the ethics committees described above and the participants provided written informed assent themselves. All methods were carried out in accordance with relevant guidelines and regulations from all institutions and research ethics boards, and are consistent with previously published work [12, 23]. Our treatment of participants according to age is consistent with the legal age of consent in Kenya, which is 18. We used the CONSORT extension for Pilot and Feasibility trials checklist downloaded from www.consort-statement.org to guide reporting, [36] see checklist in Supplementary File 1. We are aware that SCI constitute a uniquely vulnerable group. Our research group has a long history of work with SCI in Eldoret, and all aspects of this study were designed and carried out in accordance with recommendations from the Research Ethics Boards at all involved institutions, published guidelines, and our experiences from prior research [23, 37].

### Participant recruitment

Participants were recruited through ongoing convenience sampling of SCI from July 2018 to February 2020 by peer navigators (PN), who shared information about the opportunity to participate in the study with their contacts in the street community. PN, defined as persons less than 30 years of age with greater than 1 year of recent experience being street-connected, are members of the research team who have extensive experience working with the street-connected community [23]. Healthcare providers could also refer participants to the study.

### Study procedures

The pilot study of the EAT intervention ran from July 23, 2018 to February 28, 2020. Participants were eligible to receive one free hot meal per day when they presented to the study site to take their dose(s) of medication at any time between 9am and 5 pm. A pharmacy technologist distributed medications and observed doses 6 days per week, and participants on daily medications were given the 7^th^ day dose(s) to take independently. ART for HIV was available free of charge through AMPATH, and MTRH provided a waiver for all costs associated with non-HIV medications.

EAT participants could be on a variety of different medications with different dosing schedules, and the frequency and duration of their visits to the EAT site could vary. For example, participants could enrol in EAT to complete a week-long course of antibiotics or a 28-day course of PEP. Participants could also choose to visit the EAT site on a non-daily schedule, for example once per week, and be dispensed doses of medication to take independently until their next visit. Participants on medication dosed more than once per day took one observed dose during their EAT visit and were given the subsequent doses to take independently. Not all participants visited daily, and we found that supplying 25 food servings per day was adequate and minimized food waste. We offered a meal to children accompanying their parents to prevent the participant from giving food to the child instead of eating it themselves. We did not provide participants with any food to take away. We also did not provide participants with money for transportation to or from the clinic. The pilot study start and end dates were defined based on funding and team member availability. PN followed-up (in person or over the phone) with participants who missed clinic or mDOT visits and accompanied participants to health care visits if requested.

### Data collection & analysis

At enrolment, the pharmacy technologist administered a questionnaire that recorded sociodemographic information and medication prescriptions. The pharmacy technologist scanned the participants’ right thumbs with a biometric fingerprint sensor in order to generate a unique identification code to track medication dispensation and attendance (for more detail on the use of biometric finger scanners, see Braitstein et al. 2019) [12]. Participant attendance data were collected daily, checked for errors, and de-identified. Data were inputted into a REDCap database, a secure research-dedicated database developed by collaborators at Vanderbilt University. REDCap data were exported into electronic spreadsheets to carry out descriptive statistics (percentage, mean, median, confidence intervals (CI), standard deviation, and interquartile range (IQR)).

Data on date of enrolment in HIV care at AMPATH, status in HIV care at AMPATH, and viral load (VL) measurements at AMPATH prior to and during the EAT intervention were linked to participant identification codes, exported from AMPATH medical records, and de-identified. Status in HIV care at AMPATH was categorized as active in care (defined as: attended a clinic visit at an AMPATH clinic within 90 days of the expected follow-up date) or lost to follow up (LTFU) (defined as: no visit at an AMPATH clinic within 90 days of the expected follow-up date). Status in EAT was categorized as active in EAT or LTFU in EAT (defined as: 2 weeks of non-attendance at EAT and unsuccessful attempts to follow up by PN, with no return to EAT by the end of the study period). Mortality was ascertained through AMPATH clinical records and outreach by PN. Anonymized participant HIV care data were imported into electronic spreadsheets then imported into SAS 9.3. For readers interested in youth, we present outcomes among participants aged 15–24 years in the Supplementary File 2. As this was a pilot study assessing proof of concept, it was not powered to detect changes in HIV treatment outcomes. To better characterize our findings, we used McNemar’s chi-square test to calculate the difference in proportion of participants who were active in care at AMPATH, initiated ART, had a viral load measure, and were virally suppressed at their last viral load measure in the 19 months before the EAT intervention vs. the 19 months of EAT follow up. We considered the test as significant at *p* < 0.05. We limited this analysis to the participants who were enrolled in AMPATH before EAT so we had a paired sample of participants with data pre- and post-intervention. Using the Wilcoxon rank sum test, we compared time to initiation of ART and time to first episode of viral suppression among participants who were enrolled in AMPATH prior to EAT vs. enrolled in AMPATH concurrently with EAT. We considered the test as significant at *p* < 0.05.

We conducted a cost-outcome description as described by Drummond and Sculpher et al. 2015 [38]. We described total costs for 19 months of the EAT intervention as a sum of fixed and variable costs [38–41]. Fixed costs were defined as costs that do not vary with the length of program operation over the short term, for example, costs associated with setting up the EAT office. Variable costs defined as costs that vary with the length of program operation and number of participants, for example, food costs and staff salaries. We present variable costs per month per participant, calculated by dividing monthly costs (which were stable per month) by the median number of unique participants who visited the EAT site at least once per month. This analysis only included costs from the perspective of the intervention implementers (e.g. office costs, staff salaries, program supplies) and participants (e.g. travel costs). We present fixed costs and variable costs separately from transport costs because transport costs were borne by participants while other costs were paid by the study. We do not consider overhead costs or drug costs associated with EAT because these were funded by separate programs and cost estimation is beyond the scope of this pilot study. We also do not estimate costs or savings associated with downstream healthcare impacts or productivity impacts of EAT as these are beyond the scope of this pilot study. All calculations are presented in the Supplementary File 3. Costs were originally recorded in Kenyan Shillings (KSH) and converted to United States dollars (USD) using the average USD to KSH exchange rate between July 23 2018 and February 28 2020 based on the daily indicative exchange rates provided by the Central Bank of Kenya [42].

## Results

### Participant sociodemographic data

EAT enrolled 87 participants, 41 (47%) men and boys and 46 (53%) women and girls. Of the 87 participants, 41 (47%) were more than 30 years of age, 33 (38%) were between 20 and 29 years of age, 10 (12%) were between 10 and 19 years of age, and 3 (3%) were younger than 10 years of age. The mean age was 28.4 (IQR 23–34). At baseline, 47 (54%) of participants slept in a house, 20 (23%) slept on the street, 14 (16%) slept in the bases/barracks (outdoor places of congregation), and 6 (7%) stayed in a shared shelter with other SCI. Overnight, 34 (39%) participants stayed with friends, 27 (31%) stayed with a spouse/boyfriend/ girlfriend, 8 (9%) stayed alone, and 6 (7%) stayed with parents. Almost one fifth (17%) of participants were accompanied by children on their first visit to the clinic (Table 1).

**Table 1:** Participant sociodemographic data at initial EAT visit

	**Total**	**Male**	**Female**
	***N* = 87**	***N* = 41**	***N* = 46**
	**n (%)**	**n (%)**	**n (%)**
**Age**			
< 5 years	3 (3)	1 (2)	2 (4)
5 to 9 years	0	0	0
10 to 14 years	4 (5)	2 (5)	2 (4)
15 to 19 years	6 (7)	3 (7)	3 (7)
20 to 24 years	18 (21)	10 (24)	8 (17)
25 to 29 years	15 (17)	4 (10)	11 (24)
> 30 years	41 (47)	21 (51)	20 (43)
**Sleeping at night**			
In a shared shelter	6 (7)	6 (15)	0 (0)
On the street	20 (23)	10 (24)	10 (22)
Barracks/Base	14 (16)	7 (17)	7 (15)
In a house	47 (54)	18 (44)	29 (63)
**Stay with at night**			
Parent(s)	6 (7)	3 (7)	3 (7)
Other Family Member	2 (2)	1 (2)	1 (2)
Sibling	2 (2)	0	2 (4)
Friends	34 (39)	21 (51)	13 (28)
Spouse, boyfriend, girlfriend	27 (31)	10 (24)	17 (37)
Alone	8 (9)	4 (10)	4 (9)
Other	8 (9)	2 (5)	6 (13)
**Children accompanying to clinic**			
Yes	15 (17)	3 (7)	12 (26)
No	72 (83)	38 (93)	34 (74)

### Participant scheduled mDOT regimens

Of the 87 participants, 75 (86%) were living with HIV and received ART as part of their mDOT regimen. Two (2%) participants living with HIV also received tuberculosis treatment, and 58 (67%) received a course of antibiotics at some point during the EAT follow up period. Of the 12 participants not living with HIV, 3 (3%) received tuberculosis treatment, 6 (7%) received PrEP, 1 received PEP, 1 received antibiotics, and 1 received psychiatric medication (Table 2).

**Table 2:** Participant activity in the EAT program

	**Total**	**Males**	**Females**
	***N* = 87**	***N* = 41**	***N* = 46**
	**n (%)**	**n (%)**	**n (%)**
**Drug regimen at baseline**			
ART for HIV treatment	15 (17)	5 (12)	10 (22)
ART for HIV treatment + TB Treatment	2 (2)	2 (5)	0
ART for HIV treatment + antibiotic(s)	58 (67)	22 (54)	36 (78)
PrEP with or without antibiotic(s) or other medication	6 (7)	6 (15)	0
PEP with or without antibiotic(s) or other medication^a^	1 (1)	1 (2)	0
TB Treatment with or without antibiotic(s) or other medication^a^	3 (3)	3 (7)	0
Antibiotic(s) with or without other medication	1 (1)	1 (2)	0
Other medication only^a^	1 (1)	1 (2)	0
**Schedule of EAT visits**^b^			
Daily	49 (56)	27 (66)	22 (48)
Weekly	35 (40)	13 (32)	22 (48)
Inconsistent	3 (3)	1 (2)	2 (4)
**Barriers to attendance at first visit**			
No transport fare	80 (92)	35 (85)	45 (98)
Police and other law enforcement^c^	2 (2)	2 (5)	0
Other^d^	5 (6)	4 (10)	1 (2)
**Total number of visits**	5761	2712	3049
**Median # of visits (IQR)**	57 (20–132)	59 (21–131)	55 (20–133)
**Participant status at end of pilot**			
Active in EAT	58 (67)	29 (71)	29 (63)
Not active in EAT	29 (33)	12 (29)	17 (37)
**Reasons for not active in EAT**			
Lost to follow-up from EAT^e^	10 (11)	3 (7)	7 (15)
Lost to follow-up from AMPATH^f^	2 (2)	0	2 (4)
Deceased	2 (2)	0	2 (4)
Relocated	10 (11)	4 (10)	6 (13)
In prison	5 (6)	5 (12)	0

*PrEP* pre-exposure prophylaxis

*PEP* post-exposure prophylaxis

^a^Other medications included olanzapine, insulin, and paracetamol

^b^Schedule of EAT visits is how often SCI were supposed to attend EAT to receive medications. The schedule was determined by the pharmacy technologist and the participant in accordance with the participant’s medication regimen and the participant's ability to attend EAT, e.g. some participants said they would come daily to collect medications, whereas some said they would prefer to come weekly and collect the entire weeks’ dose of pills

^c^Other law enforcement include county officials, Askari

^d^Other barriers to attendance included not feeling well, disruptions related to substance use (e.g. had used alcohol and were unable to come), and familial responsibilities (e.g. taking care of a young child)

^e^Lost to follow up from EAT defined as: 2 weeks of non-attendance at EAT and unsuccessful attempts to follow up by PN, with no return to EAT by the end of the study period

^f^Lost to follow up from AMPATH defined as: no visit at an AMPATH clinic within 90 days of the expected follow-up date

Approximately half of the enrolled participants were scheduled for daily EAT visits (56%), with the rest scheduled for weekly visits (40%) or other schedules (3%) according to their medication prescription and preferences.

### Participant activity and attendance at EAT

From July 23 2018 to February 28 2020, EAT recorded 5761 visits. Most participants did not adhere to the visit schedules assigned at the first visit and attendance was irregular. The median number of visits per individual participant over the study period was 57 (IQR 20–132). The median number of unique participants who visited the study site at least once per month from August 2018 to February 2020 was 42 (IQR 39–50). The median number of visits to the study site per month in the same period was 316 (IQR 249–374). At the first visit, the most common barrier to attendance was having money for transportation to the clinic where EAT was located. Other barriers to attendance are listed in Table 2.

### Engagement in HIV care and treatment prior to enrolment in EAT

**Fig. 1:**
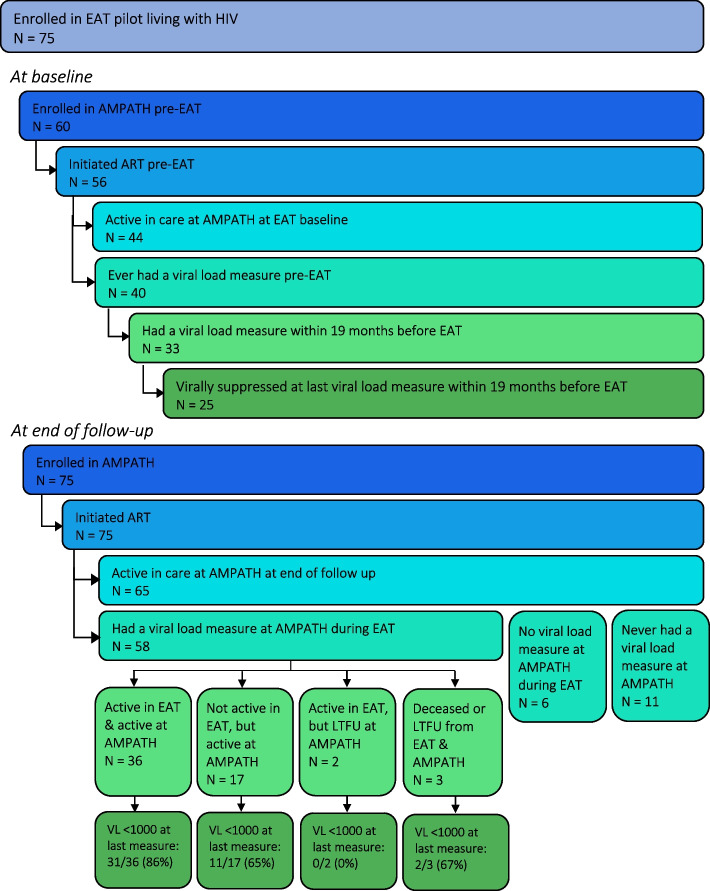
Viral suppression is defined as VL<1000 (as per Kenya Ministry of Health and AMPATH Guidelines).  ART: Antiretroviral therapy; VL: Viral load measure

Prior to enrolment in EAT, 60 (80%, 95% CI 71–89%) out of 75 participants living with HIV had been enrolled in HIV care at AMPATH. Of the 60 participants previously enrolled in HIV care, at enrolment in EAT 56 (93%, 95% CI 87–100%) had initiated ART and 44 (73%, 95% CI 62–85%) were active in care at AMPATH, 40 (67%, 95% CI 55–79%) had ever had a viral load measure, 33 (55%, 95% CI 42–68%) had a viral load measure in the 19 months prior to enrolment in EAT, and 25 (42% 95% CI 29–54%) were virally suppressed at their last viral load measure in the 19 months prior to enrolment in EAT. (VL < 1000 copies/mL) (Fig. 1).

### Engagement in HIV care and treatment at end of follow-up

At the end of follow-up, 75 (100%) participants living with HIV were enrolled in HIV care at AMPATH, all had initiated ART, and 65 (87%, 95% CI 79–94%) were active in care at AMPATH. Fifty-eight (77%, 95% CI 68–87%) participants had a viral load measure after starting EAT, of whom 44 (59%, 95% CI 48–70%) met the AMPATH definition of virally suppressed (VL < 1000 copies/mL) at their last viral load measure during the study period (Fig. 1). Among the 60 participants who were enrolled in AMPATH before EAT, at the end of follow up compared to baseline, there was a 15% increase in proportion of participants active in care at AMPATH (*p* = 0.039) and a 28% increase in the proportion of participants who had a viral load measure (*p* = 0.001). There was a 21% increase in the proportion of participants suppressed at their last viral load measure within the 19 months of EAT follow up compared to the 19 months before EAT enrollment (*p* = 0.012) (Table 3).

**Table 3:** Outcomes at baseline vs. end of follow up among participants who enrolled in AMPATH prior to EAT (a pre/post comparison)

	**At EAT baseline *N* = 60 n (%, 95% CI)**	**At end of EAT follow up *N* = 60 n (%, 95% CI)**	***P*-Value**^**a**^
Active in care at AMPATH	44 (73, 62–85)	53 (88, 80–96)	**0.039**
Initiated ART	56 (93, 87–100)	60 (100)	1.000
Had a viral load measure^a^	33 (55, 42–68)	50 (83, 74–93)	** < 0.001**
Virally suppressed at most recent viral load measure^a^	25 (42, 29–54)	38 (63, 51–76)	**0.012**

^a^Within the 19 months before EAT, for equivalence with duration of EAT follow-up

*P*-value associated with McNemar’s Chi-square test statistic

Bold values indicate statistical significance

### Time to ART initiation and first episode of viral suppression among participants who initiated ART prior to enrolment in EAT vs. after enrolment in EAT﻿

Table 4 presents HIV treatment outcomes at end of follow up stratified by whether participants enrolled in HIV care before EAT or concurrently with enrolment in EAT. Median time to ART initiation from enrolment in AMPATH was 6 months among participants who enrolled in care pre-EAT vs. 0 months among participants who enrolled in care concurrently with EAT (*p* < 0.001). Median time to first episode of viral suppression from initiation of ART was 11 months among participants who enrolled in care pre-EAT vs. 6 months among participants who enrolled in care concurrently with EAT (*p* = 0.003).

**Table 4:** End of follow up HIV treatment outcomes and last known viral load, participants stratified by sex and enrolment pre-EAT or concurrently with EAT

	**SCI living with HIV**	**Enrolled in HIV Care Pre-EAT**			**Enrolled in HIV Care Concurrently with Enrolment in EAT**			***P*-value**
	**Total**^**a**^** N = 75**	**Males**^**a**^** N = 21**	**Females**^**a**^** N = 39**	**Total**^**a**^** N = 60**	**Males**^**a**^** N = 8**	**Females**^**a**^** N = 7**	**Total**^**a**^** N = 15**	
**ART Regimen**								
** First line**	66	17	34	51	8	7	15	
** Second line**	9	4	5	9	0	0	0	
**Viral load measure ever?**								
** Yes**	64	19	37	56	4	4	8	
** No**	11	2	2	4	4	3	7	
**Viral load measure during EAT?**								
** Yes**	58	16	34	50	4	4	8	
** No**	17	5	5	10	4	3	7	
**Viral load at final measure during the study period**^**a**^	***n* = 58**	*n* = 16	*n* = 34	***n* = 50**	*n* = 4	*n* = 4	***n* = 8**	
** < 200 copies / ml**	40	10	24	34	4	2	6	
** < 1000 copies / ml**	4	1	3	4	0	0	0	
** > 1000 copies / ml**	14	5	7	12	0	2	2	
**Median time (months) to ART initiation from enrolment in care (IQR)**	3 (0–34)	6(0–39)	6(0–35)	6(0–38)	0	0	0	** < 0.001**
**Median time (months) to first episode of viral suppression from ART initiation (IQR)**^b^	9(6–25)	7(4–54)	11(8–30)	11(7–38)	6(6–6)	5(5–6)	6(5–6)	**0.003**

^a^Numbers in the three rows below total to the value denoted by “n”, the number of participants who had a viral load measure during the study period

^b^Comparison of median time using the Wilcoxon rank sum test

Bold values indicate statistical significance

### Costs

The total cost of the EAT intervention over the 19-month period, including all fixed costs and variable costs, was $57,448.64. The fixed costs were $3623.04. The total cost of setting up the EAT office was $2902.72 and contributed the largest proportion (80%) to the fixed costs. For a detailed breakdown of fixed costs, see Table 5. The variable costs per month excluding travel were $2677.62. Each month, a median of 42 unique participants visited the EAT site at least once, giving an estimated variable cost per participant per month of $63.75. The estimated travel costs for participants were $0.49 per return fare on local transport, which gives an estimated $155.31 in travel costs per month based on a median of 316 visits per month. For a detailed breakdown of costs, see Table 5 and Supplementary File 3.

**Table 5:** Costs

Fixed Costs	Costs (USD)	Costs as % of Total
Staff training (7 days)	$209.18	6%
Community mobilization activities	$294.89	8%
Office equipment	$2902.72	80%
Program supplies^a^	$216.25	6%
**Total fixed costs**	**$3623.04**	**100%**
**Variable Costs (per month)**		
Social worker, full time	$412.85	15%
Pharmaceutical technologist, full time	$766.72	29%
1 Pharmaceutical technologist on attachment, full time	$294.89	11%
1 Project coordinator, 0.5 full time equivalent	$393.19	15%
2 Peer Navigators, 0.2 full time equivalent each	$58.98	2%
Other operating costs^b^ (excluding food)	$161.21	6%
Food costs^c^	$589.78	22%
**Total variable cost (per month)**	**$2677.62**	**100%**
**Monthly variable cost per participant**^d^	**$63.75**	
**Total transportation costs per month**^**e**^	**$155.31**	
**Total costs for EAT intervention, including all fixed and variable costs and transport costs**^**f**^	**$57,448.64**	

^a^Program supplies include pill boxes, a water filter, microwave, and drinking cups

^b^Other operating costs included office supplies, phone, and internet

^c^For a fixed purchase of 25 servings of food per day, 6 days per week, 4 weeks per month

^d^Calculated by dividing total monthly costs by the median number of unique participants who visited the EAT site at least once per month

^e^Calculated based on the median number of visits to the EAT site per month (316) assuming each of those trips cost $0.49 USD (the actual cost of a return fare on local transport in USD, converted from KSH using the methods described above in this manuscript)

^f^Total costs calculated for 19 months of the EAT intervention

## Discussion

Our pilot study provides proof of concept that EAT, a combination intervention providing mDOT, food, and peer navigation services, was feasible to implement among SCI in an urban setting in Kenya and can support SCI’s engagement in HIV care and achievement of viral suppression. We also identified factors to guide implementation of trials of EAT at a larger scale or in different contexts. The estimated cost for 19 months of the EAT intervention was $57,448.64 USD.

Our study demonstrates that EAT can feasibly be implemented with this unique population and within the existing HIV care infrastructure, and provides preliminary evidence that EAT can support SCI’s engagement in HIV care and achievement of viral suppression. This pilot study was not designed to detect significant changes in HIV treatment outcomes. However, we observed that by the end of follow up all participants had initiated ART; the proportion of participants active in HIV care increased from 59% to 87%; and the proportion of participants who met the AMPATH definition of virally suppressed increased compared to the 19 months before the EAT intervention, from 33% to 59%. Among participants who were enrolled in care prior to EAT baseline, there were statistically significant increases in the proportion who were active in care at AMPATH and who had a viral load measure at end of follow up compared to baseline. There was also a statistically significant increase in the proportion of participants who were virally suppressed at their last viral load measure within the 19 months of EAT follow up compared to within the 19 months before EAT enrollment. The proportions of participants who were active in care and initiated ART are comparable to results of other interventions among populations at high risk of HIV in Kenya, for example, a program for female sex workers living with HIV where by end of follow up, 79% of participants were engaged in care and 73% were on ART [43]. Although our sample size is small, the proportion of participants virally suppressed at their last viral load test is comparable to published rates of viral suppression among adults and children in various settings across Kenya (ranging from 39.7% among a rural residents of another western county, to 51% and 60% respectively among children and adults in Uasin Gishu) [24, 44–46]. The proportion of participants virally suppressed after EAT is also similar to other interventions trialed among other sub-Saharan African populations at high risk of HIV, including female sex workers, [47] and adolescents [48, 49]. Although our findings suggest that participants who enrolled in HIV care concurrently with EAT had a statistically significantly shorter time to initiation of ART and to the first episode of viral suppression compared to participants who enrolled in HIV care before EAT, we cannot attribute this change to EAT alone, because guidelines for initiating ART have changed over time and we did not control for heterogeneity between the two groups. Additionally, EAT’s preliminary successes may be partly attributable to its unique context, embedded in AMPATH and linked to an established peer navigator program and adolescent health centre [23, 50]. However, based on our evidence that EAT can feasibly be implemented in this unique population and shows promise in improving HIV outcomes, future research should include controlled trials powered to evaluate whether EAT can improve adherence to ART and viral suppression and how EAT performs relative to other interventions.

One major goal of this proof of concept study was to identify implementation challenges or unexpected factors that could guide implementation of future trials. First, we found that although most participants were on drug regimens requiring at least daily dosing, most participants did not adhere to the dosing schedules assigned at their first visit. Irregular visit timing may be related to competing demands on SCI’s time, including work, [51] or barriers like costs of transportation. Some SCI may not need or want daily meals or treatment observation. Improvements in rates of viral suppression during EAT even though many participants did not attend daily may reflect success of the other components of EAT that supported adherence, like follow up from PN after a missed medication refill. These findings are in line with evidence that interventions using non-daily visits to encourage adherence and interventions using PN can improve HIV treatment outcomes [29, 52]. Future studies could focus on exploring how different combinations of elements in the EAT intervention function to meet participants’ needs. We also recommend that future trials of EAT explore whether (or for whom) a daily, weekly, or drop-in mDOT schedule would be most effective, and fund participants’ transport to reduce barriers to attendance. Finally, although EAT was originally conceived as an intervention for street-connected youth and eligibility was extended to adults for ethical and practical reasons, over half of the participants living with HIV in this pilot study were above the age of 30. Previous studies in our setting have focused on street-connected youth, [12, 53, 54] and the high number of adults who enrolled was unexpected, indicating a need for more research on HIV among adults who self-identify as street-connected in Eldoret.

The total cost of EAT over 19 months was $57,448.64. Fixed costs were $3623.04 USD. Variable costs were $2677.62 per month and $63.75 per participant per month, estimated using the median number of participants who visited at least once per month. Published cost estimates for non-food interventions that support ART adherence in low-resource settings range widely, from $33.00 USD for a 6-week mDOT intervention to $14.75 USD per visit for a peer-based intervention [52]. Cost estimates for nutrition interventions also vary widely, from $15-$50 per participant per month for monthly ration baskets or food dispersion [55–58]. Costs in our study may be comparatively high because EAT combined behavioral, biomedical, and structural adherence supports. Our operating costs also include some food waste, because food orders were fixed at 25 servings per day, while the number of attendees was sometimes below 25. Operating costs could be reduced by reducing food waste, achieving economies of scale, or outside of a research context where some staffing costs would be eliminated. New literature suggests that nutrition supplementation or DOT may only be cost-effective in unique populations at high risk of severe malnutrition or nonadherence to ART [59–61], which may support the use of EAT among SCI. Future trials of EAT powered to detect statistically significant changes in HIV outcomes should be paired with cost-effectiveness evaluations that account for a more complete scope of costs (including overhead costs, drug costs, and downstream healthcare costs and savings) and compare the costs of EAT to other interventions or treatment as usual for SCI.

This pilot study of the EAT intervention has a number of limitations. First, this pilot study was not powered to detect significant differences in outcomes pre- and post- intervention, and not designed assess the effectiveness of EAT compared to a control group. We did not include a direct measure of ART adherence and regular viral load measures were not required. SCI are highly transient, so our ability to follow up with participants and maintain consistent attendance was limited, although improved by the use of PN. Participants also self-identified as street-connected, so it is possible some participants did not genuinely meet our eligibility criteria for duration of street-involvement, however, ethically and practically we were limited in our ability to verify the exact duration of street involvement, and this limitation was mitigated by use of PN who know the street community well. Interpretation of our inferential statistical analyses are limited because we did not adjust for confounding, and furthermore, interpretation of differences in median time to initiation ART and to the first episode of viral suppression are limited by the fact that guidelines around initiating ART have changed over the years. Monthly operating cost estimation per participant may underestimate the true cost per participant because the actual number of participants who attended EAT every day is lower than the median number with at least one visit per month. Furthermore, we do not compare the costs of EAT to costs of any other intervention, so this study in isolation cannot guide decision making around EAT’s relative cost-effectiveness. The pilot study also has a number of strengths. Investigators were able to draw on long-standing relationships with the community of SCI in Eldoret, which likely supported engagement. It showed that the EAT intervention was feasible within the existing infrastructure, which suggests possibilities for further study and scale. Potential sources of funding for future trials of EAT might include NGOs, local governments and healthcare agencies, and research funding bodies. EAT also offers opportunities for novel partnerships, for example, between healthcare and local food or agricultural businesses who could supply meals. Anecdotally, when the pilot ended, the restaurant which operated beside the Rafiki Center voluntarily continued to provide free meals to SCI attending the clinic for ART because they observed how much it helped to stabilize and support the participating SCI. (Unfortunately, about one month later, in March 2020, the restaurant closed because of the COVID-19 pandemic.) Ultimately, EAT, if scaled, may help support SCI’s human right to an adequate standard of living, including adequate food and medical care, as set out by the Universal Declaration of Human Rights [62].

## Conclusion

Our pilot study provided proof of concept that EAT, a combination intervention providing mDOT, food, and peer navigation services, was feasible to implement and may support engagement in HIV care and achievement of viral suppression among SCI living with HIV in an urban setting in Kenya. Future EAT trials should consider a adopting a non-daily schedule for mDOT and funding participants’ travel to reduce barriers to attendance. Future research should include controlled trials powered to assess whether EAT can improve adherence to ART and viral suppression, how EAT performs relative to other interventions, and whether EAT can be feasibly implemented with SCI in other settings.

### Supplementary Information

**Additional file 1.**

** Additional file 2.** 

** Additional file 3.**

## Data Availability

The datasets generated and/or analysed during the current study are not publicly available because they contain information that could compromise participant privacy and confidentiality but are available from the corresponding author on reasonable request.

## Abbreviations

AMPATH: Academic Model Providing Access to Healthcare
ART: Antiretroviral therapy
EAT: Enabling Adherence to Treatment intervention
ESYHI: Engaging Street Youth in HIV Interventions study
HIV: Human immunodeficiency virus
IQR: Interquartile range
KSH: Kenyan Shillings
LTFU: Lost to follow up
mDOT: Modified directly observed therapy
MTRH: Moi Teaching and Referral Hospital
PEP: Post-exposure prophylaxis
PN: Peer navigators
PrEP: Pre-exposure prophylaxis
SCI: Street-connected individuals
USD: United States dollars
VL: Viral load

## References

[1] United Nations Special Rapporteur on the right to adequate housing. Report of the Special Rapporteur on adequate housing as a component of the right to an adequate standard of living, and on the right to non-discrimination in this context. 2015. https://undocs.org/A/HRC/31/54. Accessed 2 December 2 2021.

[2] Tipple G, Speak S (2005). Definitions of homelessness in developing countries. Habitat Int.

[3] Institute of Global Homelessness. A global framework for understanding and measuring homelessness. 2015. https://ighhub.org/resource/global-framework-understanding-and-measuring-homelessness. Accessed 2 December 2021.

[4] Fazel S, Geddes JR, Kushel M (2014). The health of homeless people in high-income countries: descriptive epidemiology, health consequences, and clinical and policy recommendations. The Lancet.

[5] United Nations Committee on the Rights of the Child. General Comment No. 21 (2017) on children in street situations. 2017. https://www.streetchildren.org/resources/general-comment-no-21-2017-on-children-in-street-situations/. Accessed 2 December 2021.

[6] United Nations Committee on the Rights of the Child. Protection and promotion of the rights of children working and/or living on the street. 2012. https://www.streetchildren.org/resources/protection-and-promotion-of-the-rights-of-children-working-andor-living-on-the-streets/. Accessed 2 December 2021.

[7] Woan J, Lin J, Auerswald C (2013). The health status of street children and youth in low- and middle-income countries: a systematic review of the literature. J Adolesc Health.

[8] Asibey BO, Conroy E, Marjadi B (2020). Health problems and healthcare service utilisation amongst homeless adults in Africa-a scoping review. BMC Public Health.

[9] Liu M, Hwang SW (2021). Health care for homeless people. Nat Rev Dis Primers.

[10] Kibel M, Pierzchalski J, Gorfinkel L, Embleton L, Ayuku D, Hogg R (2020). Standardized mortality ratios between street-connected young people and the general age-equivalent population in an urban setting in Kenya from 2010 to 2015. Glob Health Action.

[11] Lohrmann GM, Botha B, Violari A, Gray GE (2012). HIV and the urban homeless in Johannesburg. South Afr J HIV Med.

[12] Braitstein P, Ayuku D, DeLong A, Makori D, Sang E, Tarus C (2019). HIV prevalence in young people and children living on the streets. Kenya Bull World Health Organ.

[13] Roy É, Haley N, Leclerc P, Cédras L, Weber AE, Claessens C (2003). HIV incidence among street youth in Montreal, Canada. AIDS.

[14] Braitstein P, DeLong A, Ayuku D, Ott M, Atwoli L, Galárraga O (2021). Association of care environment with HIV incidence and death among orphaned, separated, and street-connected children and adolescents in western Kenya. JAMA Netw Open.

[15] United Nations Secretary-General. Affordable housing and social protection systems for all to address homelessness: report of the Secretary General. 2019. https://digitallibrary.un.org/record/3840349?ln=en. Accessed 2 December 2021.

[16] Rotich E, Mbai I, Marete I, Yego F, Veny MB, Lelong BA (2012). Being homeless: reasons and challenges faced by affected women. Afr J Midwifery Womens Health.

[17] Embleton L, Shah P, Gayapersad A, Kiptui R, Ayuku D, Braitstein P (2020). Characterizing street-connected children and youths' social and health inequities in Kenya: a qualitative study. Int J Equity Health.

[18] Aronstam AL, M'Mbone F, Kwena Z, Lin JS, Bukusi EA, Auerswald CL (2013). Stigma and the street: Sending communities' perceptions of street boys in Luanda District, Kenya. J Adolesc Health.

[19] Embleton L, Shah P, Apondi E, Ayuku D, Braitstein P. If they had a place to live, they would be taking medications: a qualitative study identifying strategies for engaging street-connected young people in the HIV prevention-care continuum in Kenya. Under re-review at the J Int AIDS Soc. 2022. 10.1002/jia2.26023 PMC10237327 37267115

[20] Chop E, Duggaraju A, Malley A, Burke V, Caldas S, Yeh PT (2017). Food insecurity, sexual risk behavior, and adherence to antiretroviral therapy among women living with HIV: a systematic review. Health Care Women Int.

[21] Becker N, Cordeiro LS, Poudel KC, Sibiya TE, Sayer AG, Sibeko LN (2020). Individual, household, and community level barriers to ART adherence among women in rural Eswatini. PLoS ONE.

[22] Embleton L, Shah P, Apondi E, Ayuku D, Braitstein P (2023). “If they had a place to live, they would be taking medication”: a qualitative study identifying strategies for engaging street-connected young people in the HIV prevention-care continuum in Kenya. J Int AIDS Soc.

[23] Shah P, Kibel M, Ayuku D, Lobun R, Ayieko J, Keter A (2019). A pilot study of "Peer Navigators" to promote uptake of HIV testing, care and treatment among street-connected children and youth in Eldoret, Kenya. AIDS Behav.

[24] National AIDS Control Council. Kenya HIV County Profiles. National AIDS and STI Control Program (NASCOP); 2016. https://nacc.or.ke/wp-content/uploads/2016/12/Kenya-HIV-County-Profiles-2016.pdf. Accessed 2 December 2021.

[25] UNAIDS. Combination HIV prevention: Tailoring and coordinating biomedical, behavioural and structural strategies to reduce new HIV infections. A UNAIDS discussion paper. 2010. https://www.unaids.org/sites/default/files/media_asset/JC2007_Combination_Prevention_paper_en_0.pdf. Accessed 2 December 2021.

[26] Hwang SW, Burns T (2014). Health interventions for people who are homeless. The Lancet.

[27] Naranbhai V, Abdool Karim Q, Meyer-Weitz A (2011). Interventions to modify sexual risk behaviours for preventing HIV in homeless youth. Cochrane Database Syst Rev.

[28] Hart JE, Jeon CY, Ivers LC, Behforouz HL, Caldas A, Drobac PC (2010). Effect of directly observed therapy for highly active antiretroviral therapy on virologic, immunologic, and adherence outcomes: a meta-analysis and systematic review. J Acquir Immune Defic Syndr.

[29] Scanlon ML, Vreeman RC (2013). Current strategies for improving access and adherence to antiretroviral therapies in resource-limited settings. HIV AIDS (Auckl).

[30] Remme JH, Adam T, Becerra-Posada F, D'Arcangues C, Devlin M, Gardner C (2010). Defining research to improve health systems. PLoS Med.

[31] Research NNIfHaC. Proof of concept for applications to the EME program. Accessed from: https://www.nihr.ac.uk/documents/proof-of-concept/19909.

[32] Peters DH, Tran NT, Adam T. Implementation research in health: a practical guide: World Health Organization; 2013.

[33] Kenya National Bureau of Statistics. Volume III: Distribution of population by age, sex, and administrative units. 2019 Kenya Population and Housing Census. III: Nairobi; 2019.

[34] Mercer T, Gardner A, Andama B, Chesoli C, Christoffersen-Deb A, Dick J (2018). Leveraging the power of partnerships: Spreading the vision for a population health care delivery model in western Kenya. Glob Health.

[35] Kibel M, Shah P, Ayuku D, Makori D, Kamaara E, Choge E (2019). Acceptability of a pilot intervention of voluntary medical male circumcision and HIV education for street-connected youth in western Kenya. J Adolesc Health.

[36] Eldridge SM, Chan CL, Campbell MJ, Bond CM, Hopewell S, Thabane L (2016). CONSORT 2010 statement: extension to randomised pilot and feasibility trials. BMJ.

[37] Embleton L, Ott MA, Wachira J, Naanyu V, Kamanda A, Makori D (2015). Adapting ethical guidelines for adolescent health research to street-connected children and youth in low- and middle-income countries: a case study from western Kenya. BMC Med Ethics.

[38] Drummond MF, Sculpher MJ, Claxton K, Stoddart GL, Torrance GW. Methods for the economic evaluation of health care programmes: Oxford university press; 2015.

[39] Wilson-Barthes M, Chrysanthopoulou SA, Atwoli L, Ayuku D, Braitstein P, Galarraga O (2021). Cost-effectiveness of care environments for improving the mental health of orphaned and separated children and adolescents in Kenya. J Ment Health Policy Econ.

[40] Galárraga O, Shah P, Wilson-Barthes M, Ayuku D, Braitstein P (2018). Cost and cost-effectiveness of voluntary medical male circumcision in street-connected youth: findings from an education-based pilot intervention in Eldoret, Kenya. AIDS Res Ther.

[41] Galárraga O, Wamai RG, Sosa-Rubí SG, Mugo MG, Contreras-Loya D, Bautista-Arredondo S (2017). HIV prevention costs and their predictors: evidence from the ORPHEA Project in Kenya. Health Policy Plan.

[42] Central Bank of Kenya. Key CBK Indicative Exchange Rates USD - KSH: Central Bank of Kenya. Accessed from: https://www.centralbank.go.ke/rates/forex-exchange-rates/. Accessed 2 December 2021.

[43] Bhattacharjee P, Musyoki HK, Becker M, Musimbi J, Kaosa S, Kioko J (2019). HIV prevention programme cascades: insights from HIV programme monitoring for female sex workers in Kenya. J Int AIDS Soc.

[44] Maina EK, Mureithi H, Adan AA, Muriuki J, Lwembe RM, Bukusi EA (2020). Incidences and factors associated with viral suppression or rebound among HIV patients on combination antiretroviral therapy from three counties in Kenya. Int J Infect Dis.

[45] Humphrey JM, Genberg BL, Keter A, Musick B, Apondi E, Gardner A (2019). Viral suppression among children and their caregivers living with HIV in western Kenya. J Int AIDS Soc.

[46] Maman D, Zeh C, Mukui I, Kirubi B, Masson S, Opolo V (2015). Cascade of HIV care and population viral suppression in a high-burden region of Kenya. AIDS (London, England).

[47] Kerrigan D, Mbwambo J, Likindikoki S, Davis W, Mantsios A, Beckham SW (2019). Project Shikamana: community empowerment-based combination HIV prevention significantly impacts HIV incidence and care continuum outcomes among female sex workers in Iringa, Tanzania. J Acquir Immune Defic Syndr.

[48] Zanoni BC, Sibaya T, Cairns C, Lammert S, Haberer JE (2017). Higher retention and viral suppression with adolescent-focused HIV clinic in South Africa. PLoS ONE.

[49] Mburu M, Guzé MA, Ong'wen P, Okoko N, Moghadassi M, Cohen CR (2019). Evaluating the effectiveness of the HIV adolescent package of care (APOC) training on viral load suppression in Kenya. Public Health.

[50] Embleton L, Di Ruggiero E, Logie CH, Ayuku D, Braitstein P (2020). Piloting an evidence-based intervention for HIV prevention among street youth in Eldoret. Kenya Int J Public Health.

[51] Sorber R, Winston S, Koech J, Ayuku D, Hu L, Hogan J (2014). Social and economic characteristics of street youth by gender and level of street involvement in eldoret, Kenya. PLoS One.

[52] Penn AW, Azman H, Horvath H, Taylor KD, Hickey MD, Rajan J (2018). Supportive interventions to improve retention on ART in people with HIV in low- and middle-income countries: a systematic review. PLoS One.

[53] Embleton L, Wachira J, Kamanda A, Naanyu V, Ayuku D, Braitstein P (2016). Eating sweets without the wrapper: perceptions of HIV and sexually transmitted infections among street youth in western Kenya. Cult Health Sex.

[54] Embleton L, Wachira J, Kamanda A, Naanyu V, Winston S, Ayuku D, et al. “Once you join the streets you will have to do it": Sexual practices of street children and youth in Uasin Gishu County, Kenya Adolescent Health. Reprod Health. 2015;12 (1)(106):1–11. 10.1186/s12978-015-0090-z PMC4647324 26573581

[55] Tirivayi N, Koethe JR, Groot W (2012). Clinic-based food assistance is associated with increased medication adherence among HIV-infected adults on long-term antiretroviral therapy in Zambia. J AIDS Clin Res.

[56] Martinez H, Palar K, Linnemayr S, Smith A, Derose KP, Ramírez B (2014). Tailored nutrition education and food assistance improve adherence to HIV antiretroviral therapy: evidence from Honduras. AIDS Behav.

[57] Benzekri NA, Sambou JF, Tamba IT, Diatta JP, Sall I, Cisse O (2019). Nutrition support for HIV-TB co-infected adults in Senegal, West Africa: A randomized pilot implementation study. PLoS ONE.

[58] Mamlin J, Kimaiyo S, Lewis S, Tadayo H, Jerop FK, Gichunge C (2009). Integrating nutrition support for food-insecure patients and their dependents into an HIV care and treatment program in Western Kenya. Am J Public Health.

[59] McCabe CJ, Goldie SJ, Fisman DN (2010). The cost-effectiveness of directly observed highly-active antiretroviral therapy in the third trimester in HIV-infected pregnant women. PLoS ONE.

[60] Uthman RT, Sutton AJ, Jackson LJ, Uthman OA (2018). Does directly administered antiretroviral therapy represent good value for money in sub-Saharan Africa? a cost-utility and value of information analysis. PLoS ONE.

[61] Koethe JR, Marseille E, Giganti MJ, Chi BH, Heimburger D, Stringer JS (2014). Estimating the cost-effectiveness of nutrition supplementation for malnourished, HIV-infected adults starting antiretroviral therapy in a resource-constrained setting. Cost Eff Resour Alloc.

[62] United Nations General Assembly (1948). Universal declaration of human rights. UN General Assembly.

