# Supplementary material for: Enabling Adherence to Treatment (EAT): a pilot study of a combination intervention to improve HIV treatment outcomes among street-connected individuals in western Kenya
Source: BMC Health Serv Res. 2023 Nov 30;23:1331. doi: 10.1186/s12913-023-10215-1 (PMC10691070; doi:10.1186/s12913-023-10215-1)
Supplement: Supplementary file 2 — Additional file 2. [file 12913_2023_10215_MOESM2_ESM.docx]

Supplementary file II

EAT outcomes among youth

Young people (defined as participants aged 15-24 years) were a key population of interest for this program. Prior to EAT baseline, 12 out of 16 participants aged 15-24 years living with HIV had enrolled in HIV care at AMPATH, 12 had initiated ART, 9 were active in care at AMPATH when they enrolled in EAT, and 4 were virally suppressed at their last viral load measure prior to baseline (VL<1000 copies/mL). At end of follow-up, all 16 participants aged 15-24 years living with HIV had initiated ART, 13 were active in care at AMPATH, 13 had a viral load measure after starting EAT, and 10 met the AMPATH definition of virally suppressed (VL<1000 copies) at their last viral load measure during the study period.
